# Age-Associated Donor-Site Selection Patterns in Pediatric Maxillary Microvascular Reconstruction: A 14-Year Single-Center Experience

**DOI:** 10.3390/jcm15103824

**Published:** 2026-05-15

**Authors:** Dominika Lech, Robert Maksymowicz, Jeremi Matysek, Aleksandra Strzelecka, Cyprian Strączek, Marcin Kozakiewicz, Łukasz Krakowczyk, Krzysztof Dowgierd

**Affiliations:** 1 Department of Clinical Pediatrics, Head and Neck Surgery Clinic for Children and Young Adults, University of Warmia and Mazury, 10-709 Olsztyn, Poland; 2 Department of Maxillofacial Surgery, Medical University of Lodz, 113 Żeromskiego Str., 90-549 Lodz, Poland; 3 2nd Department of Oncologic Surgery, Maria Sklodowska Curie Memorial National Cancer Center, 44-100 Gliwice, Poland

**Keywords:** pediatric maxillary reconstruction, microvascular free flap, donor-site selection, iliac crest flap, medial femoral condyle flap, fibula free flap, Cordeiro classification, flap survival

## Abstract

**Background:** Reconstruction of maxillary defects in pediatric patients presents unique challenges related to craniofacial growth, functional rehabilitation, and long-term treatment planning. Although microvascular free-flap reconstruction is established in pediatric head and neck surgery, maxilla-specific data remain limited. This study aimed to describe donor-site selection patterns in pediatric maxillary microvascular reconstruction and to explore their relationship with patient age. **Methods:** This retrospective observational study included pediatric patients (aged 1–18 years) who underwent microvascular free-flap reconstruction of the maxilla between August 2011 and September 2025 at a single tertiary referral center. Clinical variables included age, defect characteristics, reconstruction timing, donor-site, and total flap loss. Donor-site selection patterns were analyzed in relation to patient age using nonparametric statistical methods. **Results:** Fifty-five patients were included. Overall flap survival was 81.82% (45/55). Donor-site selection demonstrated a significant association with patient age (*p* < 0.05). Younger children were more frequently reconstructed using soft-tissue flaps, whereas osseous flaps were more commonly used in older children and adolescents. No significant relationship was found between age and defect extent or reconstruction timing. **Conclusions:** In this single-center experience, pediatric maxillary reconstruction followed an age-adapted pattern, with soft-tissue flaps preferentially used in younger patients and osseous flaps more frequently selected in older children. These findings reflect differences in reconstructive priorities across developmental stages and support individualized planning in pediatric maxillary reconstruction. Age alone was not associated with total flap loss; however, this analysis was limited by the small number of flap loss events.

## 1. Introduction

Reconstruction of maxillary defects in pediatric patients remains one of the most demanding problems in craniofacial surgery. Unlike adults, children are still undergoing craniofacial growth and dental development, so reconstructive planning must address not only immediate restoration of form and function, but also preservation of growth potential, future dental rehabilitation, and long-term esthetic outcomes [1,2]. Previous clinical series have demonstrated high flap survival rates and acceptable complication profiles in this population [3,4,5]. As microsurgical techniques and perioperative care have advanced, the focus has gradually shifted from feasibility toward more comprehensive planning and long-term outcomes in the growing patient [2].

In this context, maxillary defects require separate consideration. Many studies on pediatric jaw reconstruction analyze maxillary and mandibular defects together [1,6,7], which limits conclusions specific to this anatomical region. This distinction is important, as these defects present unique anatomical and functional challenges, including oral–nasal separation, midfacial contour, orbital support in selected cases, and the potential for subsequent dental rehabilitation.

Existing literature on pediatric microvascular reconstruction has largely focused on flap survival, complication rates, and donor-site morbidity, particularly for vascularized bone flaps such as the free fibula flap [8,9,10]. While these data are valuable, they provide limited insight into how reconstructive decisions are adapted to the specific requirements of this region in growing patients.

In adult practice, several classification systems and reconstructive frameworks have been developed to guide flap selection according to defect extent and surgical objectives [11,12,13,14,15,16]. Among them, the Cordeiro classification remains one of the most widely used systems for describing maxillectomy defects and structuring treatment planning [11]. However, these frameworks were designed for skeletally mature patients and do not incorporate the developmental stage, which is a key factor in younger populations.

In children, reconstructive priorities may change with age. In early childhood, the primary objective may be reliable defect closure and restoration of soft-tissue contour, whereas in older patients greater emphasis may be placed on skeletal reconstruction and the creation of a foundation for long-term functional rehabilitation. Despite the clinical relevance of this age-adapted perspective, it has not been systematically characterized in cohorts focused specifically on this region.

In our previous analysis of pediatric maxillofacial microvascular reconstruction, defects of the maxilla were associated with the highest rate of flap loss among the anatomical sites studied [17]. The reasons for this observation remain unclear, and it is not known whether age contributes to reconstructive decision-making or microsurgical outcomes in this subgroup.

Therefore, further region-specific data are needed. The primary aim of the present study was to describe our single-center experience in pediatric maxillary microvascular reconstruction, with particular emphasis on age-adapted patterns of donor-site selection. We also sought to evaluate whether age was associated with total flap loss. Secondary objectives included assessment of the relationship between age and defect extent in oncologic cases, as well as reconstruction timing.

## 2. Materials and Methods

### 2.1. Study Design

This retrospective observational study included pediatric patients who underwent microvascular free-flap reconstruction of the maxilla between August 2011 and September 2025 at the Division of Maxillofacial Surgery for Children and Young Adults, Head and Neck Clinic, Regional Specialized Children’s Hospital in Olsztyn, Poland.

### 2.2. Patient Selection

Eligible patients were 1–18 years old at the time of surgery and underwent maxillary reconstruction using a microvascular free flap. Both osseous and soft-tissue free flaps were included. Cases were identified from institutional surgical records. Only patients with complete electronic medical documentation were eligible for inclusion. Patients with incomplete records were excluded.

A total of 59 patients were initially screened. Four patients were excluded due to incomplete documentation. The final study cohort consisted of 55 consecutive patients who underwent 55 reconstructive procedures.

### 2.3. Data Collection

Clinical data were extracted from electronic health records and entered into a structured database created for this study. Data extraction was performed retrospectively from operative reports, pathology reports, and inpatient documentation.

The following variables were recorded:

- Age at the time of surgery
- Sex
- Etiology of the defect
- Extent of maxillary defect according to the Cordeiro classification (applied to oncological cases only)
- Reconstruction timing (primary vs. secondary in oncological cases)
- Donor-site
- Occurrence of total flap loss

Primary reconstruction was defined as immediate reconstruction performed during ablative tumor surgery. Secondary reconstruction was defined as reconstruction performed after prior ablative surgery. Total flap loss was defined as complete flap necrosis requiring surgical removal of the flap. Follow-up data were collected from electronic medical records with a minimum observation period of 6 months for all patients. All data were anonymized prior to statistical analysis.

### 2.4. Age Categorization

Age was analyzed both as a continuous and categorical variable. For categorical analyses, patients were grouped into four predefined age categories:

- ≤5 years
- 6–10 years
- 11–15 years
- >15 years

These categories were selected to reflect clinically relevant developmental stages and to assess potential threshold effects in flap selection and outcomes.

### 2.5. Surgical Decision-Making and Operative Management

Flap selection was determined by the operating surgical team on the basis of patient-specific and defect-specific considerations. Decisions were made by experienced surgeons within a multidisciplinary clinical setting. Although no formalized protocol was applied throughout the study period, flap choice reflected a consistent reconstructive approach shaped by developmental stage, defect characteristics, reconstructive goals, and accumulated institutional experience.

In younger children, reconstructive priorities were more often focused on reliable defect closure, restoration of soft-tissue contour, and limitation of treatment burden at the donor-site. In older children and adolescents, greater emphasis was placed on restoration of the maxillary framework and on creating conditions for long-term functional rehabilitation, including future dental and prosthetic reconstruction. Final flap selection remained individualized in each case.

All procedures were performed simultaneously by two coordinated surgical teams: one responsible for resection and the other for microsurgical reconstruction, including flap harvest, inset, and microvascular anastomosis. The number of participating surgeons varied according to case complexity.

### 2.6. Statistical Analysis

Statistical analysis was performed using STATGRAPHICS Centurion 19 (StatPoint Technologies, Tulsa, OK, USA). Continuous variables are presented as mean ± standard deviation (SD) and median values. Categorical variables are presented as frequencies and percentages. Because age distribution was non-normal and group sizes were unequal (including groups with single observations), nonparametric statistical methods were applied. Comparisons of age across multiple donor-site categories and Cordeiro classification groups were performed using the Kruskal–Wallis test. When statistically significant differences were identified, post hoc pairwise comparisons were conducted using Dunn’s test. Comparisons between two independent groups (e.g., flap survival vs. total flap loss; primary vs. secondary reconstruction) were performed using the Mann–Whitney U test. Groups containing a single observation were included in descriptive analysis but excluded from inferential comparisons where appropriate. Flap survival rate was calculated as the proportion of flaps not resulting in total flap loss. All statistical analyses were interpreted cautiously because of the exploratory character of the study and the unequal size of several subgroups. A two-sided *p*-value < 0.05 was considered statistically significant.

## 3. Results

The study cohort consisted of 55 patients who underwent microvascular free-flap reconstruction of the maxilla. There were 32 females (58.18%) and 23 males (41.82%). The largest age subgroup comprised patients older than 15 years (n = 23; 41.82%), followed by those aged 11–15 years (n = 20; 36.36%). Patients aged 6–10 years accounted for 7 cases (12.73%), and 5 patients (9.09%) were 5 years old or younger.

With regard to etiology, oncological defects were the most common indication for reconstruction (n = 29; 52.73%), followed by congenital defects (n = 21; 38.18%), iatrogenic defects (n = 4; 7.27%), and traumatic defects (n = 1; 1.82%). The iliac crest was the most frequently used donor-site (n = 23; 41.82%), followed by the fibula (n = 13; 23.64%), medial femoral condyle (n = 9; 16.36%), and anterolateral thigh flap (n = 8; 14.54%). Patella and radius flaps were each used in one patient (1.82%). Overall flap survival was 81.82% (45/55), whereas total flap loss occurred in 10 patients (18.18%). Detailed demographic and clinical characteristics are presented in Table 1.

### 3.1. Age and Donor-Site Selection

Differences in patient age across donor-site categories are summarized in Table 2. The overall mean age of the cohort was 13.65 ± 4.94 years, with a median age of 15.00 years. Patients reconstructed with the anterolateral thigh flap were the youngest subgroup, with a mean age of 6.38 ± 5.58 years and a median age of 5.00 years. In contrast, patients reconstructed with osseous flaps were generally older: the mean age was 14.17 ± 4.00 years for the iliac crest group, 14.92 ± 2.75 years for the fibula group, and 17.67 ± 0.71 years for the medial femoral condyle group. The single patient reconstructed with a patella flap was 6 years old, and the single patient reconstructed with a radius flap was 15 years old.

**Figure 1:**
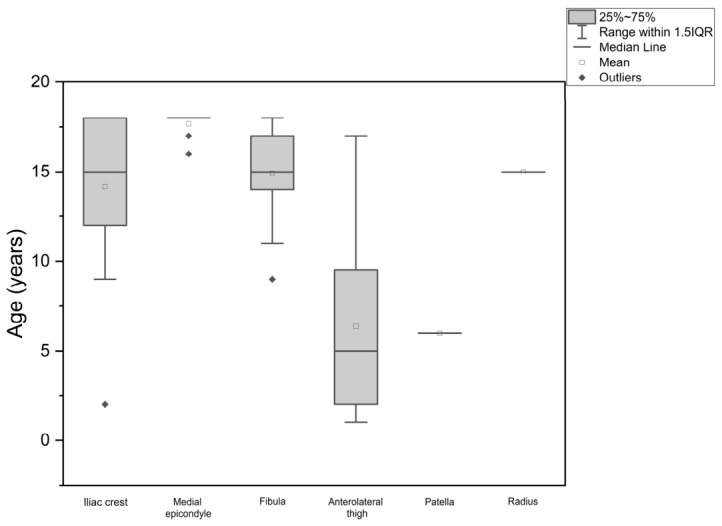
Box plot showing patients’ age distribution at each donor-site.

After excluding single-case donor-site categories, a Kruskal–Wallis test showed a significant difference in age distribution across the four donor-site categories (H(3) = 20.17, *p* < 0.05). Post hoc pairwise analysis with Dunn’s correction demonstrated a statistically significant difference between the medial femoral condyle and anterolateral thigh groups (Z = 4.44, *p* < 0.05). Overall, the observed distribution was consistent with a clinically distinct age-related pattern, in which younger children more often underwent reconstruction with soft-tissue flaps, whereas older children and adolescents more frequently received osseous flaps. The age distribution across donor-site groups is illustrated in Figure 1.

### 3.2. Age and Cordeiro Classification in Oncologic Cases

Among the 29 patients with oncological defects, the distribution of age according to Cordeiro classification is shown in Table 3. Patients with class I defects had a mean age of 10.81 ± 5.36 years and a median age of 11.50 years. In class II defects, the mean age was 15.20 ± 1.64 years and the median was 15.00 years. Patients classified as IIIA had a mean age of 7.86 ± 4.95 years and a median age of 7.00 years. One patient with a class IIIB defect was aged 12 years.

**Figure 2:**
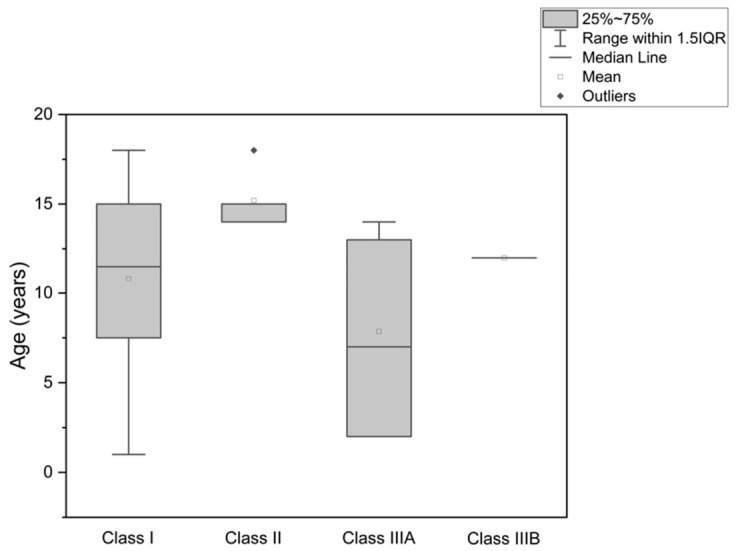
Box plot showing the patients’ age distribution for each maxillary defect class according to the Cordeiro classification.

Comparison of age across oncologic defect classes using the Kruskal–Wallis test did not show a statistically significant difference (H(3) = 6.92, *p* = 0.07). Thus, within this oncologic subgroup, no significant association was observed between age and defect extent as defined by the Cordeiro classification. The age distribution across oncologic defect classes is presented in Figure 2.

### 3.3. Age and Total Flap Loss

The relationship between patient age and the occurrence of total flap loss is summarized in Table 4. Among patients without total flap loss, the mean age was 13.42 ± 5.28 years and the median age was 15.00 years. In the group with total flap loss, the mean age was 14.70 ± 2.98 years and the median age was also 15.00 years.

**Figure 3:**
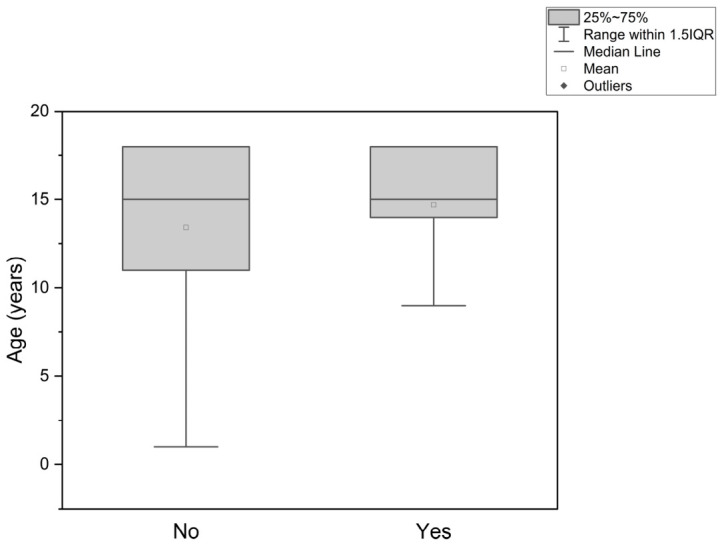
Box plot showing the patients’ age distribution for flap status (No—no total flap loss occurred; Yes—total flap loss occurred).

A Mann–Whitney U test showed no statistically significant difference in age between patients with flap survival and those with total flap loss (U = 214.50, *p* = 0.82). These findings indicate that age was not associated with the occurrence of total flap loss in this cohort. The corresponding age distribution is shown in Figure 3.

### 3.4. Age and Flap Survival Within Individual Donor-Site Groups

Age distributions according to flap survival status within individual donor-site groups are presented in Table 5. In the iliac crest group, successful flap transfer was achieved in 18 patients, while total flap loss occurred in 5 patients, corresponding to a flap survival rate of 78.26%. The mean age was 14.17 ± 4.26 years in the survival group and 14.20 ± 3.27 years in the flap loss group; this difference was not statistically significant (U = 41.5, *p* = 0.82). In the medial femoral condyle group, 8 flaps survived and 1 total flap loss was observed, yielding a flap survival rate of 88.89%. Comparative analysis was not performed because the flap loss subgroup contained only a single patient. In the fibula group, 9 flaps survived and 4 were lost, corresponding to a flap survival rate of 69.23%. The mean age was 15.11 ± 2.85 years in the survival subgroup and 14.50 ± 2.89 years in the flap loss subgroup, with no significant difference between them (U = 20, *p* = 0.81). All anterolateral thigh, patella, and radius flaps survived.

### 3.5. Age and Reconstruction Timing in Oncologic Cases

**Figure 4:**
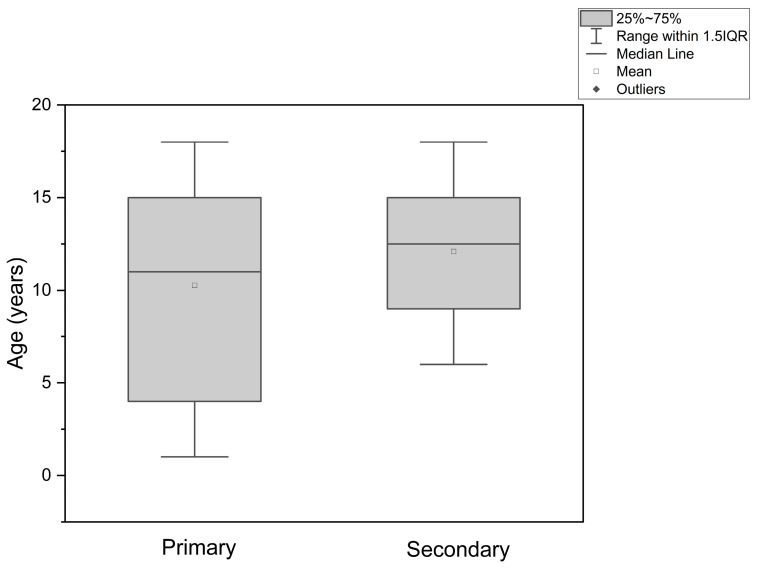
Box plot displaying the age distribution among patients who underwent primary and secondary oncological reconstruction.

Among oncologic cases, primary reconstruction was performed in 19 patients and secondary reconstruction in 10 patients. The mean age in the primary reconstruction group was 10.26 ± 5.75 years, with a median of 11.00 years. In the secondary reconstruction group, the mean age was 12.10 ± 3.78 years and the median age was 12.50 years. Although a Mann–Whitney U test was used to compare age between these groups, as shown in Figure 4, no statistically significant difference was observed.

### 3.6. Age, Donor-Site Selection, and Etiology

Table 6 summarizes age and donor-site selection according to oncological and non-oncological etiologies. In oncological cases, the iliac crest was used in 13 patients, fibula in 7, anterolateral thigh in 8, and patella in 1. In non-oncological cases, iliac crest reconstruction was performed in 10 patients, medial femoral condyle in 9, fibula in 6, and radius in 1.

Among oncological cases, after excluding single-case donor-site categories, patient age differed significantly across donor-site groups (H(2) = 8.34, *p* < 0.05). Post hoc analysis with Dunn correction demonstrated a significant difference between the fibula and anterolateral thigh groups (Z = 2.85, *p* = 0.027). In the non-oncological subgroup, no statistically significant difference in age across donor-site groups was found (H(2) = 3.43, *p* = 0.18). Overall, the oncologic subgroup showed the clearest age-related variation in donor-site selection.

### 3.7. Age and Flap Survival According to Etiology

Table 7 summarizes age and flap survival status in oncological and non-oncological cases. In oncological defects, 22 reconstructions were successful and 7 resulted in total flap loss, corresponding to a flap survival rate of 75.86%. The mean age was 10.00 ± 5.44 years in patients with flap survival and 13.71 ± 2.93 years in those with total flap loss. In non-oncological defects, 23 reconstructions were successful and 3 resulted in total flap loss, giving a flap survival rate of 88.46%. The mean age was 16.70 ± 2.14 years in the survival group and 17.00 ± 1.73 years in the flap loss group.

Mann–Whitney U testing showed no statistically significant age difference between patients with flap survival and those with total flap loss, either in oncological cases (U = 108.5, *p* = 0.11) or in non-oncological cases (U = 32.00, *p* = 0.85). These findings indicate that, within both etiological subgroups, age was not significantly associated with flap loss.

## 4. Discussion

The present study suggests that donor-site selection in pediatric maxillary microvascular reconstruction follows a clinically meaningful age-adapted pattern. Younger children were more often reconstructed with soft-tissue flaps, whereas osseous flaps were used more frequently in older children and adolescents. In contrast, patient age was not associated with total flap loss, defect extent according to the Cordeiro classification, or the timing of oncologic reconstruction. Taken together, these findings indicate that, in this cohort, age may have been more closely related to reconstructive strategy than to microsurgical outcome.

This pattern is consistent with everyday clinical reasoning in pediatric reconstruction. In younger children, the immediate priority is often reliable defect closure and restoration of soft-tissue contour, whereas in older patients’ greater emphasis is placed on reconstruction of the maxillary framework and preparation for long-term functional rehabilitation. The observed age-related pattern may also reflect fundamental principles of craniofacial growth and skeletal maturation. In pediatric patients, midfacial development depends on continuous bone remodeling, sutural growth, dentoalveolar development, and functional interactions between the maxilla, mandible, skull base, and occlusion [1]. Disruption of these mechanisms, either at the recipient site or through donor-site morbidity, may result in long-term functional and esthetic consequences. Consequently, reconstructive priorities differ across developmental stages. In younger children, preservation of growth potential, reliable separation of the oral and nasal cavities, restoration of soft-tissue contour, and minimization of donor-site morbidity are critical, which may favor the use of soft-tissue flaps. In contrast, in older children and adolescents, restoration of maxillary skeletal support becomes increasingly important for maintaining midfacial projection, supporting occlusal relationships, and enabling future dental or prosthetic rehabilitation, thereby supporting the use of osseous flaps. Additionally, technical limitations of bone-containing flaps in younger patients, including reduced bone stock, size mismatch, and uncertainty regarding future implant placement, may further influence reconstructive decision-making [1].

Although the literature does not define a strict age-based algorithm for pediatric maxillary reconstruction, similar strategies have been described. Garfein et al. reported successful use of both soft-tissue and osseous flaps in pediatric midface reconstruction with generally favorable functional and esthetic outcomes [2], while Upton noted that the fibula is commonly preferred for reconstruction in growing children and adolescents [18]. At the same time, age should not be interpreted as an independent determinant of flap selection. Reconstructive decision-making in children is inherently multifactorial and depends on defect characteristics, etiology, anticipated functional demands, and long-term rehabilitation goals. Within this framework, age functions primarily as a practical indicator of developmental stage and helps guide priorities rather than acting as an isolated deciding factor.

The observed distribution of flap types also corresponds to the broader literature on maxillary reconstruction. As emphasized by Andrades et al. and Iyer and Thankappan, reconstruction of this region requires balancing several competing objectives, including speech, swallowing, facial contour, and structural support [12,13]. Costa et al. further highlighted the importance of bone-containing flaps when restoration of the skeletal framework is required [14]. In pediatric patients, these reconstructive aims must additionally be considered in the context of ongoing growth and future rehabilitation. Genden et al. emphasized the importance of preserving growth potential while achieving satisfactory reconstructive outcomes [1], and Alkureishi et al. reported favorable long-term results with low donor-site morbidity after pediatric free-flap reconstruction [19]. Although growth-related outcomes and donor-site morbidity were not assessed in the present study, both are likely to influence flap selection in clinical practice.

Although our findings support the general feasibility of free tissue transfer in children, the observed survival rate—81.82%—was lower than that reported in the literature. Previous studies have demonstrated higher success rates, including 99.8% in the series by Upton and Guo [18], 96.4% in the meta-analysis by Markiewicz et al. [20], and 95.4% in pediatric oncologic patients reported by Starnes-Roubaud et al. [21]. In our previous work, maxillary reconstruction was identified as the anatomical site with the highest rate of flap loss within pediatric maxillofacial reconstruction. The present study was designed in part to investigate whether patient age could explain this observation. However, the current findings do not support this hypothesis, as age was not associated with total flap loss in this cohort.

A more plausible explanation may lie in the inherent complexity of this anatomical region. Reconstruction of the maxilla often involves simultaneous management of multiple functional and structural components, including separation of the oral and nasal cavities, restoration of facial contour, and, in selected cases, provision of structural support and a basis for future dental rehabilitation. This distinguishes it from many other reconstructive sites and supports considering it as a separate subgroup within pediatric microsurgery [12,14]. At the same time, our institution functions as a tertiary referral center and receives a considerable number of complex cases, including patients with extensive defects, prior treatment, or unfavorable local conditions. This combination of cases likely contributed to the lower flap survival observed in the present cohort.

Direct comparison with previously published pediatric microsurgical series is challenging because most available studies include heterogeneous head and neck defects, combined mandibular and maxillary reconstructions, or predominantly mandibular cases. In contrast, the present cohort was restricted to maxillary reconstruction, which represents a smaller and anatomically more complex subgroup. To our knowledge, this is one of the largest maxilla-specific pediatric microvascular reconstruction cohorts reported to date. Therefore, the lower flap survival rate observed in our series may partly reflect differences in cohort composition, defect complexity, and referral patterns rather than directly comparable institutional outcomes. However, because no comparative institutional or temporal analysis was performed, this explanation remains interpretative.

The relationship between age and flap survival remains unclear in the literature. Liu et al. identified the 5–9-year age group as a potential risk factor for flap failure in a large pediatric head and neck cohort, including a predominance of mandibular reconstructions, although other variables such as donor-site and defect location were not associated with outcome [4]. The difference between their findings and ours likely reflects differences in study populations, as their cohort included a wide range of head and neck reconstructions, whereas the present study focuses specifically on the maxilla. Our results should therefore be interpreted as maxilla-specific rather than as evidence that age is universally unrelated to flap survival in pediatric microsurgery.

The negative findings in the oncologic subgroup are also noteworthy. Age was not significantly associated with defect extent according to the Cordeiro classification, suggesting that the observed differences in flap selection were not clearly explained by defect size in this cohort. However, this result approached statistical significance and should be interpreted cautiously, particularly given the limited subgroup size. In addition, classification systems such as Cordeiro’s, while useful for anatomical description, do not account for developmental stage or long-term rehabilitation, which are central considerations in pediatric reconstruction [11,12,13]. Similarly, age was not associated with the choice between primary and secondary oncologic reconstruction. This suggests that reconstruction timing is more likely determined by tumor-related and treatment-related factors than by chronological age. Previous analyses have emphasized multidisciplinary planning and demonstrated the feasibility of free-flap reconstruction even in oncologic pediatric patients [2,21].

This study has several limitations that should be considered when interpreting the findings. Its retrospective, single-center design introduces the potential for selection bias and limits the generalizability of the results. The statistical analysis was limited to univariable methods. Multivariable analysis was not performed due to the relatively small sample size and the presence of multiple subgroups with low event counts. Therefore, the results, particularly in subgroup analyses, should be interpreted cautiously. Reconstructive decision-making was not standardized and likely reflected a combination of patient-specific factors, surgeon preference, defect characteristics, and evolving clinical practice. Given the 14-year study period, temporal changes in surgical technique, perioperative management, and reconstructive philosophy, as well as potential surgeon- or team-related effects, could not be formally assessed and may have influenced flap selection. Therefore, the observed age-related pattern of donor-site selection should be interpreted within the context of a tertiary referral center managing complex pediatric maxillary defects. Finally, the study focused primarily on flap survival as the primary outcome and did not include long-term functional, esthetic, or growth-related outcomes, nor donor-site morbidity assessment.

## 5. Conclusions

This study provides one of the few maxilla-specific analyses of age-adapted reconstructive strategy in pediatric microvascular surgery. In this single-center cohort, donor-site selection demonstrated a consistent age-related pattern. Soft-tissue flaps were more commonly used in younger children, whereas osseous flaps were selected more frequently in older patients and adolescents. This association likely reflects surgeon-dependent prioritization of reconstructive goals across developmental stages rather than a standardized age-based algorithm. Patient age was not associated with total flap loss. However, because the number of flap loss events was limited, this finding should be interpreted cautiously and should not be considered evidence that age has no influence on flap survival. Future multicenter studies with larger cohorts, multivariable analysis, and long-term functional, esthetic, growth-related, and donor-site outcomes are needed to better define reconstructive decision-making in pediatric maxillary reconstruction.

## Figures and Tables

**Table 1:** Summary of demographic and clinical data of the studied patients.

	Number of Patients	Percentage of the Total Number of Patients
Sex		
Female	32	58.18%
Male	23	41.82%
Age group		
≤5 years old	5	9.09%
6–10 years old	7	12.73%
11–15 years old	20	36.36%
>15 years old	23	41.82%
Etiology		
Congenital	21	38.18%
Oncological	29	52.73%
Traumatic	1	1.82%
Iatrogenic	4	7.27%
Donor-site		
Iliac crest	23	41.82%
Medial femoral condyle	9	16.36%
Fibula	13	23.64%
Anterolateral thigh	8	14.54%
Patella	1	1.82%
Radius	1	1.82%
Tumor		
Not applicable	26	47.27%
Benign	14	25.46%
Malignant	15	27.27%
Cordeiro classification group		
Not applicable	26	47.27%
I	16	29.10%
II	5	9.09%
IIIA	7	12.72%
IIIB	1	1.82%
Primary or secondary oncological reconstruction		
Not applicable	26	47.27%
Primary	19	34.55%
Secondary	10	18.18%
Total flap loss		
No	45	81.82%
Yes	10	18.18%
Total	55	100%

**Table 2:** Summary statistics for patients’ age by their donor-site.

Type of Donor-Site	Number of Patients	Mean Age (Standard Deviation) [Years]	Median Age [Years]
Iliac crest	23	14.17 (±4.00)	15.00
Medial femoral condyle	9	17.67 (±0.71)	18.00
Fibula	13	14.92 (±2.75)	15.00
Anterolateral thigh	8	6.38 (±5.58)	5.00
Patella	1	6.00 (±0)	6.00
Radius	1	15.00 (±0)	15.00
Total	55	13.65 (±4.94)	15.00

**Table 3:** Summary statistics for patients’ age by extent of oncological defect according to the Cordeiro classification.

Cordeiro Classification	Number of Patients	Mean Age (Standard Deviation) [Years]	Median Age [Years]
Class I	16	10.81 (±5.36)	11.50
Class II	5	15.2 (±1.64)	15.00
Class IIIA	7	7.86 (±4.95)	7.00
Class IIIB	1	12.00 (±0)	12.00
Total	29	10.90 (±5.16)	12.00

**Table 4:** Summary statistics for patients’ age by the occurrence of total flap loss.

Total Flap Loss Occurrence	Number of Patients	Mean Age (Standard Deviation) [Years]	Median Age [Years]
No	45	13.42 (±5.28)	15.00
Yes	10	14.70 (±2.98)	15.00
Total	55	13.65 (±4.94)	15.00

**Table 5:** Summary statistics for patients’ age and survivability of free flap from donor-sites.

Status of Free Flap	Number of Patients	Mean Age (Standard Deviation) [Years]	Median Age [Years]
Iliac crest			
Survived	18	14.17 (±4.26)	15.00
Total flap loss	5	14.20 (±3.27)	15.00
Medial femoral condyle			
Survived	8	17.63 (±0.75)	18.00
Total flap loss	1	18.00 (n/a)	18.00
Fibula			
Survived	9	15.11 (±2.85)	15.00
Total flap loss	4	14.50 (±2.89)	14.50
Anterolateral thigh			
Survived	8	6.38 (±5.58)	5.00
Total flap loss	0	n/a	n/a
Patella			
Survived	1	6.00 (n/a)	6.00
Total flap loss	0	n/a	n/a
Radius			
Survived	1	15.00 (n/a)	15.00
Total flap loss	0	n/a	n/a
Total	55	13.65 (±4.94)	15.00

n/a—non-applicable.

**Table 6:** Summary statistics for patients’ age and selection of donor-site in oncological and non-oncological etiologies.

Type of Donor-Site	Number of Patients	Mean Age (Standard Deviation) [Years]	Median Age [Years]
Oncological etiology			
Iliac crest	13	12.15 (±4.16)	13.00
Fibula	7	14.43 (±2.07)	14.00
Anterolateral thigh	8	6.38 (±5.58)	5.00
Patella	1	6.00 (n/a)	6.00
Non-oncological etiology			
Iliac crest	10	16.80 (±1.55)	18.00
Medial femoral condyle	9	17.67 (±0.71)	18.00
Fibula	6	15.50 (±3.51)	17.00
Radius	1	15.00 (n/a)	15.00
Total	55	13.65 (±4.94)	15.00

n/a—non-applicable.

**Table 7:** Summary statistics for patients’ age and flap survival status in oncological and non-oncological etiologies.

Flap Status	Number of Patients	Mean Age (Standard Deviation) [Years]	Median Age [Years]
Oncological etiology			
Survived	22	10.00 (±5.44)	14.00
Total flap loss	7	13.71 (±2.93)	11.50
Non-oncological etiology			
Survived	23	16.70 (±2.14)	18.00
Total flap loss	3	17.00 (±1.73)	18.00
Total	55	13.65 (±4.94)	15.00

## Data Availability

The data presented in this study are available on request from the corresponding author.
